# Philosophical Medicine

**Published:** 1857-01-01

**Authors:** 


### Art. V.—PHILOSOPHICAL MEDICINE.

Most modern medical authors have concerned themselves greatly with questions and inquiries which are fragmentary and shifting. Libraries have been written upon special modes and niceties in treatment, phases of disease, and theories subtending the *modus operandi* of evanescent remedies. While much of the medical literature of the age results thus, on the whole, in a painful and disappointing reiteration of principles which are unsettled, and opinions which are often merely academical, the philosophy of medicine has come to be virtually ignored. The young physician is not taught to think systematically for himself, but rather asked to observe what others have mapped out for him. He is educated in the confused belief that there are facts belonging to theories, and theories somehow flowing from facts, not by any necessary process of induction, but on the authority of certain doctrines, and of names, which he is bound to revere. He looks in vain, if he looks at all, for something in modern medicine to satisfy the reasoning powers. He misses a consistent and complete philosophy of the whole subject. Glimpses there are in various works of the day—works, many of them, in other respects of the highest value—of philosophical methods of observation and research in medicine; but, fragmentary and shifting, these serve rather to confuse the wanderer in search of a system. Whether from a too exclusive education, or from some other cause, the author-teachers of our day do not seem to recognise the intellect as a main agent of research in medicine.

Never was it more the fashion to decry empiricism—to undervalue the aids of simple observation and experience,—and yet by a seeming, though not a real contradiction, never perhaps has medicine been so *material* in its doctrines and in its practice as it is now. Histology, chemical analysis, and minutiæ in physical diagnosis, are held up to the attention of enthusiastic disciples, as the chief of professional attainments—the indispensable, almost the exclusive, means of research; as, certainly, they are the pillars of that bare scientific medicine which, under the meaningless title of *Rational*, has latterly vaunted itself, perhaps too rashly. To this too exclusive education of the senses, a theoretical medicine has been added, which wants value, because it does not include—nor is it even based upon—any consistent scheme of observation capable of being applied, as a whole, to practical medicine. Thus, the essential characteristics of the medicine of the modern schools are, the pursuit of extreme physical analysis on the one hand, and on the other a tendency to rash mental deductions. What seems wanting is a consistent, and withal a complete, philosophy of medicine. But is such a philosophical system possible? — and if possible, is it likely to be valuable as a guide to an improved, and an improving, treatment of disease? In now attempting to answer these questions, on the whole, satisfactorily in the affirmative, we may, not inaptly, glance back at certain phases through which our individual medical faith has passed, as illustrative of what we have just been saying respecting the incompleteness of modern medical doctrines, and their insufficiency, as a connected system, to satisfy the intellect.

Like most of those who early imbibe the notion that there is an academical road to medical learning, we commenced with a vast contempt for empirical knowledge in general, and a silent distrust of data derivable from observation and experience. Proportionately enamoured of theories of disease, and full of sympathy for all attempts at demonstrating the *modus operandi* of therapeutical remedies, we had faith, likewise, in those niceties in physical diagnosis, which are still the crowning boast of some clinical authorities. We were, in fact, a faithful disciple of that still reigning school, which, imitating our seniors, without inquiring into the meaning of the term—we were fond of designating "Rational Medicine." It was at the bedside (but not in the wards of an hospital) that we first became distrustful of theory, and soon detected its insufficiency, and even its danger, as a guide in the treatment of disease. There, also, it was not long ere we were forced to embrace, what seemed to us then the distressing conviction, that minutiæ in physical diagnosis are often impracticable, and, when practicable, generally useless in practice. As to the probability of demonstrating the *modus operandi* of therapeutical remedies, that ambitious notion soon became dreamy, and finally vanished, as the baseless fabric of a vision. For, assuming that such an inquiry was not in itself the pursuit of a shadow, it soon became evident that, administered as drugs usually are, under every variety of condition, promiscuously and in combination, to avoid fallacy, an entirely original series of carefully guarded observations and experiments would first of all have to be instituted on every drug, and probably also on various doses of every drug separately;—a gigantic undertaking, and on the whole impracticable. Having reached this state of comparative negation—having lost much of our original faith in medicine, and our respect for it as a professed scientific system—by a natural reaction we took refuge in a creed of empiricism, and embraced the belief, once so distasteful, that there is no rational foundation possible for medicine, save what rests on a wise observation and experience. In that instructive region which lies behind us—a region still too little explored, we think, by many of our modern teachers—we found not a little to reconcile us to such a faith. Great men lived before Epaminondas, and we never could sympathize with that tone of affected contempt or pity with which it has latterly been the mode to speak of the ancient physicians We never could bring ourselves to believe that those painstaking observers were the wilful and ignorant empirics they are so often represented. We have good evidence that many of them were guided by what corresponded in their day to enlightened experience. Defective on some vital points, as their knowledge unquestionably was, the accuracy of their descriptions of diseases which are still common, and their conceptions of pathological conditions in relation to prognosis, are, after all, wonderful monuments of an intellectual medicine, which, with all our modern physical aids to the senses, we should not be ashamed to venerate. Obscurity veils their modes of therapeutical treatment; but it is to them we owe most of the drugs on which physicians, after many vain new therapeutical flights, are compelled mainly to rely at this hour. To us, then, it seemed that a modern empiricism—founded like the ancient, on observation and experience, taking advantage besides of the advances since made in various departments of medical research—was the only consistent faith, in the present state of that healing *art*, which, assuredly, has not yet put on the veritable garb of *science*. Such was—such, partly, still is—our medical faith; and, unsatisfactory as in some respects it may have been, we felt it, and we still consider it, safer than that in which we set out. As we have said, we had caught glimpses, here and there, of philosophical design; but these were only fragmentary, and failed to do more than suggest what we think we have at length found in a work lately published*—something nearly approaching to a connected and consistent philosophy of medicine.

Such a philosophical scheme we feel bound to accept, in the mean time, as the only possible compromise between modern empiricism, and that intolerant and self-styled rationalism which seeks to impose itself on us under the mask of science. Scientific medicine, *per se*, we have found to be unsafe, chaotic, impracticable. Empirical medicine, *per se*, we have found to be, in a different direction, unsatisfying to the intellect. Of philosophical medicine, *per se*, we cannot properly speak; because, as philosophy, it includes and inculcates whatever is practicable and true in all systems. This is its distinguishing characteristic and excellence; without which it would not be a philosophical, but, like much that has preceded it, a dogmatic, or merely doctrinal medicine. If, then, philosophical medicine has any special mission, it is not that of revolutionizing, but that of reconstructing the healing art, by showing us how the materials at our disposal—whether they be derivable from observation and experience, or theory, or art, or science—can be correctly employed, all together, or each in its proper place, in the researches and in the practice of medicine. How far, in the work of Dr. Laycock, now before us, such an attempt has been successfully made, we shall endeavour to determine, by instituting a general analysis of its scheme and method.

A distinguished metaphysician (Lewes) has observed, "It is a law of the human mind that speculations on all generalities begin deductively; and the only road to truth is to begin inductively." If this be true, need we wonder that in medicine, as in philosophy, bold speculations should so often precede or anticipate that train of corrected observations on which they ought to be based; and that theories and hypotheses, so adopted, should be retained in medicine under the name of facts or truths? To obviate this fundamental error in method, philosophical medicine requires that we begin on an inductive basis; and so far from rejecting or despising observation and experience, even of the simplest kind, that we accept them as of the first value in medical research. But then, in complex inquiries such as those of medicine, there are obviously two kinds of observation, and consequently a difference in the inductions derivable from them; and likewise there are two kinds of experience. There are what may be termed a blind observation, and a simple or unenlightened experience; as well as a corrected or compared obser-

* *Lectures on the Principles and Methods of Observation and Research. By Thomas Laycock, M.D., &c., Professor of the Practice of Medicine, and of Clinical Medicine, in the University of Edinburgh.* Edinburgh, 1856.

vation, and an enlightened experience. It is on the latter that philosophical medicine leans; although it by no means denies the use, or even the value, of the former. Again, though secondary in the ranks of an inductive method of research, hypothesis and theory are quite admissible; and rightly admitted, they are of great value also in medicine. Locke, speaking of their employment in general philosophy, says, "If they are well made, they are at least great helps to the memory, and often direct us to new discoveries. But we should not take up any one too hastily, *which the mind, that would always penetrate into the causes of things, and have principles to rest on,* is very apt to do. And, at least, that the name of *principles* deceive us not, nor impose on us, by making us receive that for an unquestionable truth which is really a very doubtful conjecture." Philosophical medicine admits, therefore, not crude theory, but the combination of theory with observation and experience. Thus combined, but not otherwise, theories are, in fact, essential to medical research. But, as Bacon, in enunciating the inductive method in philosophy, was careful to point out, there are various idols, or fallacies, which are apt to beset the inquirer; and, accordingly, philosophical medicine requires that these, as they affect medical methods of research, should be thoroughly exposed, in order that they may be avoided. Perhaps, the chief theoretical fallacies here to be guarded against, are, the indefinite use of terms, the substitution of theories themselves for medical facts, and the *post hoc ergo propter hoc*, so common in etiological and therapeutical inquiries. These common fallacies of theory are capable of being detected by the test of experience; and what may be termed compound fallacies in theory, are also amenable to the same tribunal. Experience is, in fact, "the Ithuriel's spear for all hypothetical conclusions or theoretical views we may be tempted to adopt in medicine." Moreover, no true theory is possible without scientific knowledge and inquiry; science is, therefore, a necessary element in a philosophical system of medicine.

On such a foundation we may speak of medical *science*, without holding that medicine is now, or need necessarily ever attain, the rank of a true or exact science. The basis of our medical science is none other than experience; and it results that it may be practised as an *art*. Out of this arises a consideration of the methods and the aids by which medicine is to be practised. There are instrumental aids, aids to the senses, and there are aids to the intellect alone. The latter involve subjects for special consideration. As to the former, it is most important to take a correct estimate of aids to clinical research. The senses may be educated at the expense of the intellect. Microscopical research may be abused, and too great a dependence upon it is imminently dangerous, as evinced by the errors into which modern pathologists have fallen. The stethoscope, also, will have much to answer for, if it call off attention which should be directed to the general outward features of disease. Symptoms are often of great value, and there is a *physiognomy* of disease which richly deserves study. Physiognomical diagnosis, though as yet only rudimentary, is a singularly important department of practical medicine; and, when more fully developed, "*will add more to our available knowledge than physical diagnosis, in the same proportion as the reason penetrates more deeply than the senses into the nature of things.*" It is likewise of great moment to arrive at the causes of disease, by tracing, when practicable, the origin and order of etiological phenomena; that is, in fact, the order of succession of vital phenomena. Here, as in some other places, medical science presents a large gap, or hiatus, which must, in the construction of a connected philosophical system, be filled up with all convenient diligence. For instance—"We know something of the alternations, sleeping and waking, of the menstrual period, of the periodical influence of night and day, and of the seasons. Nor are we altogether ignorant of the morbid changes to which each age and sex are liable at different ages, and the like. We know, too, the periods of several forms of fever, such as the exanthemata and the intermittents. Little of this knowledge, however, has as yet entered the domain of physiological science, and even, as to pathology and prognosis, the general, or at least the accepted knowledge of critical days is hardly more applicable to clinical medicine than in the time of Galen."

Clinical examination (or diagnosis) is further to be prosecuted, in general, on the natural method; that is, by "simple observation of the phenomena of the disease, and comparison of them with one another, and with the knowledge which the practitioner has acquired of similar phenomena, either by instruction or experience;" and the disease having in this way been determined, the therapeutical diagnosis, that is, the treatment of the disease, naturally arises out of it. But here, in entering on the management and supervision of the patient, there are certain general morbid conditions to be kept in view—the various diatheses and cachexiæ. A diathesis is an innate hereditary constitution of the body; whilst a cachexia is essentially an actually existing state, which, though not hereditary, may also be acquired by the patient himself. An acquaintance with the signs and symptoms of the various diatheses and cachexiæ, in all their combinations, is of much greater moment to the treatment of disease in general than has hitherto been supposed. Our author enters upon a very careful consideration of them, which he concludes thus:— "I have dwelt at some length upon these cachexiæ, as important *notanda* when taking a case, partly because there can be no philosophical diagnosis or therapeutics without a thorough knowledge of them, but mainly because the information regarding them, in our systematic works on medicine, is scattered through many volumes, and, at best, is imperfect. What I have detailed is the result of much careful observation and thought on my own part."

Next, in prognosis, which in its turn arises out of the therapeutical diagnosis, the signification, and not the extent of phenomena, is the important element; phenomena being minute in a popular or relative sense only. Thus, the detection and calculation of critical days, and the mode of observing periodic changes in fevers and disease generally, deserve careful study. Menstrual and dentitional periods, also, are important, because significant, phenomena. Under this category comes, likewise, the observation of meteorological influences, and seasonal changes affecting diseases—departments once neglected, but the importance of which is now beginning to be appreciated. "Henceforth medical meteorology must take its place in the cycle of the medical sciences." Prognosis, thus, may be termed scientific, inasmuch as it is founded on certain laws of occurrence and recurrence; but it is also empirical, inasmuch as it is often simply dependent on observation and experience. To this scheme of clinical observation our author adds an instructive lecture, "On the Due Estimate of Treatment, and on the Management of the Case." Herein he notices certain sources of fallacy—similar to those of theory, chiefly as to cause and effect, formerly adverted to—which are apt to invade therapeutical observations, and vitiate therapeutical conclusions. In the management of the case, perhaps the chief points are, the due estimate of probabilities, and the exercise of a moral control and influence over the patient.

This completes what on an ordinary system would be (it is here only philosophically connected and arranged) a summary of clinical medicine. But an inductive scheme of medical research would not be philosophically consistent and complete, without the application to medicine of those aids also to the intellectual powers which the inductive philosophy has supplied, with the happiest results, to other branches of experimental knowledge. Hence follows a consideration of two methods, both of which are open to us in this intellectual field. First, the simple numerical method; secondly, that more extended philosophical method, which our author has termed the *analogical*. These "have each a special value, according to the nature of the questions to be solved." The *numerical method* (which in its applications to political economy is termed generally *statistics*) has, in medicine, been too much disparaged on the one hand, and over-estimated on the other—"The fallacies of other methods in medical research affect equally the numerical;" and this is especially true as regards the use of terms. All our collective terms in medicine being more or less fallacious, "the collective facts which they express are necessarily fallacious too, both as to the deductions that may be drawn from them, and the simplest information they may convey." It is, however, in the tabulation of events of a simple character that vital statistics are of most value; and one of the great objects of numerical investigation—indeed *the* knowledge specially aimed at—is "to determine the order of events, or, in other words, the relation of cause and effect." At the same time, vital statistics are liable to serious fallacies, which, as public hygiene is now a recognised branch of medicine, ought to be carefully discriminated—"It must always be remembered that, in proportion as the circumstances or events to be compared increase in number, the sources of fallacies increase in perhaps more than a geometrical proportion." Moreover, "a numerical statement may be true, as a mere fact of experience, but fallacious as premises for comparison and deduction." It is, therefore, necessary to adopt certain defined precautions in the application of the numerical method to the investigation of questions in life and organization. On the whole, this method is of limited application to the investigations of medical science; for, "inasmuch as it reduces everything to numerical ratios and expressions, it necessarily deals only with facts and observations capable of such reduction. But these are but a few of the facts of medicine." Hence the necessity, in a full philosophical scheme, for some less restricted method of medical research; a method which may extend its inquiry to all facts; which may make use of the numerical method, but as a subordinate instrument only—a method, in short, eminently comprehensive and philosophical.

In what he terms the *analogical*, or by excellence the *philosophical*, our author believes he has discovered such a method of research. And here, in noticing the crowning part of his work, we would pause to claim for Dr. Laycock the merit of decided originality. The doctrines of the so-called transcendental anatomy, since first enunciated by Oken and Von Bär, have been freely applied to abstract physiology, and may even have proved suggestive, in an incidental way, in other medical directions; but, so far as we know, our author has been the first to found upon the doctrine of the unity of structure and function of organisms a distinct and connected method, practically available in medicine. Should this method, therefore, become generally recognised, and stand the test of experience, it is not too much to say, that its author should take a place amongst those who correspond, in the historical progress of medicine, to the Newtons and the Bacons of science and philosophy. From what has gone before, it will be apparent, at least, that we have been listening to no mere medical enthusiast—still less to one superficially versed, either in science or general philosophy; we should, therefore, be all the more ready to give to this new method—which is itself, indeed, almost a distinct philosophy of medicine—a serious and respectful attention. For the present, we regret that the lecture which our author devotes to the subject must be dismissed with the same analytic brevity that has marked our notice of the preceding longer, but not more important, part of the work.

The analogical method begins by taking up a new standpoint. Whereas the systems hitherto propounded, under the title of Rational or Scientific, have—all of them admittedly or impliedly—actually started from the admission that medicine possesses no primitive fact, no primary *law;* the analogical method starts from, or upon, a great principle, which already has something more to rest on than conjecture or opinion, since it has found acceptance in physiology, but which has yet been overlooked as a guide in practical medicine. This method analyses pathology, and finds it to be pathological physiology, and, therefore, still physiology; disease being "simply a deviation from the natural order of events as to the structure and functions of the body." The great truth of human physiology is, "that man is but a link in the infinite scheme of life; and the primary or fundamental principle of life is the unity of structure and function of organisms." Now, this principle of life and organization is of unlimited application, and more especially as a guide to correct medical theory; although it need not be the starting-point of all theories, inasmuch as there are principles of more limited application which may be used under, or within, the higher one. Thus, the principle that will guide us most readily to true analogies is this simple numerical principle, "that phenomena agreeing upon one point be collated as to that point in all their relations." Then, *discovery by true analogies is always progressive.* "Just (says our author) as in the numerical method the result of one tabulation leads on to another tabulation, and its result to another, so one analogy leads on to another investigation and arrangement of phenomena and another analogy; this to anotherand so on, *ad infinitum*, or so long as the inquirer can carry on his researches and attain to new facts. The only limit, indeed, to his discoveries is to be found in his limited powers of investigation; but the intellect practised in this method will penetrate in *idea* far beyond the horizon of the demonstrable, and see more or less clearly in the far distance analogies grander and yet grander still. The principle of unity of life and organization is all comprehensive; MIND, therefore, comes within the range of its operations as well as matter. This is a grand principle, for it is pregnant with researches and results of the highest importance to man in his social, moral, and intellectual relations."

Practical examples of the conduct of an analogical investigation are next given at length. Our author then considers, lucidly, certain objections that may be urged against it, and concludes with some hints as to the uses of this analogical method. Referring our readers to the suggestive and original volume of which we are now reluctantly compelled to take leave, we have but a single sentence to add, critical of this new method of medical observation and research.

Its author terms it a philosophical method. It is eminently so: indeed, it alone deserves, as it seems to us, to be so entitled by excellence. Viewing it, however, for the present, in its theoretical aspects, we might, perhaps, venture respectfully to doubt whether that other title—the purely inductive method of research—which its author has also given to it, be equally significant of its early tendencies. As including and using the numerical method, it is doubtless inductive; but, in another light, it is not *purely* so. It is inductive *à priori,* rather than *à posteriori.* For a time, at least, such a method must proceed more or less on axioms which have still to undergo laborious, or, at least, lengthened, processes of verification. But when the principle on which it is founded comes to be generally accepted—and, with our author, we venture to predict its ultimate general acceptance—this method, then no longer new, will become more and more purely inductive. Of its probable value, of its ultimate results, of its professional acceptance, it is not for us to say much now. Like all new ideas, it has its battle still to fight, and experience does not warrant us to predict that it will be at once cordially hailed generally—as we cordially hail it—in the light of a great step towards a consistent and complete philosophy of medicine. Few men less thoroughly accomplished than our author in every branch of medical science, and versed at the same time in general philosophy, could, or would, have given it to the profession, in the face of oscillating views which it is calculated to supersede. The book we have thus carefully analysed, but very imperfectly reviewed, is not great in point of size; but it outweighs very many larger modern treatises—not merely because it dates from a chair where sat a Gregory and an Alison, but because it indoctrinates that *intellectual* medicine which modern progress begins to demand.

